# Range Dividing MIMO Waveform for Improving Tracking Performance

**DOI:** 10.3390/s21217290

**Published:** 2021-11-02

**Authors:** Eun-Hee Kim, Han-Saeng Kim, Ki-Won Lee

**Affiliations:** 1 Department of Defence System Engineering, Sejong University, 209 Neungdong-ro, Gwangjn-gu, Seoul 05006, Korea; 2 LIGNex1 Co., 207 Mabuk-ro, Giheung-gu, Yongin-si 16911, Korea; hansaeng.kim@lignex1.com (H.-S.K.); kiwon.lee@lignex1.com (K.-W.L.)

**Keywords:** MIMO radar, radar waveforms, pulse doppler radar

## Abstract

A multiple-input multiple-output (MIMO) method that shares the same frequency band can efficiently increase radar performance. An essential element of a MIMO radar is the orthogonality of the waveform. Typically, orthogonality is obtained by spreading different signals into divided domains such as in time-domain multiplexing, frequency-domain multiplexing, and code domain multiplexing. This paper proposes a method of spreading the interference signals outside the range bins of interest for pulse doppler radars. This is achieved by changing the pulse repetition frequency under certain constraints, and an additional gain can be obtained by doppler processing. This method is very effective for improving the angular accuracy of the MIMO radar for a small number of air targets, although it may have limitations in use for many targets or in high clutter environments.

## 1. Introduction

As the application field of radar systems has expanded, the number of radar sensors operating at the same frequency has been continuously increasing. Moreover, the use of the multiple-input multiple-output (MIMO) method can efficiently increase radar performance by sharing the same frequency band. There have been many studies on MIMO performance in recent years [1,2,3,4]. MIMO radars can be used to increase the virtual antenna size, which improves the angular resolution through coherent processing [5], or be used in a bistatic manner that is transmitted and received from different sites [6].

Multiple transmission methods in MIMO radar include the array-space multiple transmission method that transmits different waveforms from different arrays and synthesizes them in the receiver, and the beam-space multiple transmission method that transmits different waveforms in different directions. In all cases, because the different transmit waveforms should be received independently in the receiver, it is necessary to use signals with orthogonal characteristics. Unfortunately, because the delay of the reflective signal is specified by the target position and cannot be synchronized from the receiver in radar systems, transmit waveforms should be orthogonal for all time delays, which is almost impossible. Therefore, the transmit waveforms in MIMO radar are usually designed to minimize the cross-correlation for all time delays—called a quasi-orthogonal waveform—while minimizing the sidelobe level of the autocorrelation to improve detection performance.

Orthogonality, which removes other waveforms, is achieved by dividing the power of each waveform into the different domains rather than cancelling them. The typical methods for designing orthogonal waveforms are time division multiplexing (TDM), frequency division multiplexing (FDM), and code division multiplexing (CDM) [7,8,9]. Although the TDM method has perfect orthogonal characteristics, it has low time (i.e., energy) efficiency and may require additional processing for a moving target because the measurements are not performed simultaneously. The FDM method also has perfect orthogonal characteristics but has low spectral efficiency, and range-angle coupling occurs because of the linear relationship between the frequency and the index of the antenna element. For CDM, especially in radar systems, it is difficult to find a perfect orthogonal waveform or code domain for all delay times, as mentioned above. This paper proposes a method for dividing range bins using different pulse repetition frequencies (PRFs) for pulse doppler radars.

The signal processing of the pulse doppler radar consists of a matched filter for intra-pulse modulation and coherent integration between pulses. If the received pulse is modulated by different codes, the output of the matched filter is the sum of correlations between the matched and mismatched codes. In this first step, the signal-to-interference ratio (SIR) is improved in terms of both noise and cross-correlations, constituting the interference signal. However, because the interpulse coherency is maintained even for the mismatched codes, interference by cross-correlation is not suppressed by coherent integration processing, and only the portion by the noise is further suppressed. Therefore, if the noise level is relatively high that normally occurs within the maximum detection range of a radar, the SIR is improved by both intrapulse and interpulse processing; however, as the target comes closer, the signal and the cross-correlations increase, and the interference is dominated by cross-correlations. In this close range, SIR does not change by distance and cannot be improved beyond a certain level determined by the matched filter, and it eventually affects the angle estimation accuracy.

In this paper, we propose a novel method to further improve the SIR ratio in interpulse coherent processing and enhance the angular estimation performance of virtual arrays of a MIMO radar. The proposed method uses different PRFs and intrapulse modulation codes while retaining the MIMO beamforming condition; thus, the pulse-to-pulse coherency from different transmitters is not maintained, and cross-correlations cannot obtain inter-pulse integration gain. This method is useful for improving the accuracy when there are a small number of aerial targets, but also has limitations that are difficult to use in cluttered environments because it spreads the power of other transmit signals to the irrelevant range bins.

The intrapulse code used in this study is based on polyphase codes that are designed to optimize autocorrelations and cross-correlations. There are two well-known design methods for a polyphase code. One is the family of cyclic algorithm-new (CAN) algorithms, including stopband CAN (SCAN) and periodic CAN (PeCAN), where the objective is to minimize the sum of the cross-correlation and the sidelobe of auto correlations through a cyclical process [10,11]. The other is generalized optimization methods such as the genetic or simulated annealing (SA) method [12,13,14,15], which provides flexibility in the objective function and parameter set. We applied the SA method in this paper.

The remainder of this paper is organized as follows. Section 2 summarizes the MIMO virtual array processing and describes the proposed method. Section 3 demonstrates the performance by simulation, and the conclusions are presented in Section 4.

## 2. Basic Principles

### 2.1. MIMO Signal Processing

**Figure 1:**
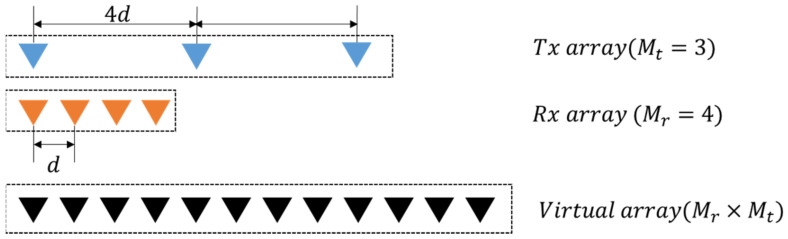
Virtual array antenna by MIMO.

For the collocated transmit and receive arrays, MIMO radars simultaneously propagate different waveforms from multiple transmit arrays and emulate a large virtual aperture with appropriate spacing. If the receive antenna is a uniform linear array (ULA) with $M_{r}$ elements arranged with intervals *d* and the transmit array is a sparse ULA with $M_{t}$ elements at $\left(M_{r}\times d\right)$ intervals, the virtual aperture is a ULA with $M_{r}\times M_{t}$ elements at *d* intervals. If $M_{t}=3$ and $M_{r}=4$, the phase difference vectors $v_{tx}\;$for the transmit, $v_{rx}$ for receiving, and v for virtual arrays are

(1) $$\begin{array}{c} v_{tx}=\left[1\;\;\;\;e^{j\left(4kd\sin\theta \right)}\;\;\;e^{j\left(8kd\sin\theta \right)}\right]\in C^{1\times M_{t}}, \end{array}$$

(2) $$\begin{array}{c} v_{rx}=\left[1\;\;\;\;e^{j\left(kd\sin\theta \right)}\;\;\;e^{j\left(2kd\sin\theta \right)}\;\;\;\;\;e^{j\left(3kd\sin\theta \right)}\;\right]\in C^{1\times M_{r}}\;, \end{array}$$

and

(3) $$v=v_{tx}⊗v_{rx}=\left[1\;\;\;\;e^{jkd\sin\theta }\;\;\;e^{j\left(2kd\sin\theta \right)}\cdots \;\;e^{j\left(11kd\sin\theta \right)}\;\right]\;\in C^{1\times \left(M_{r}\times M_{t}\right)}$$

where ⨂ is the Kronecker product, and k (=2π⁄λ) is the wave number. **v** is identical to that of a ULA composed of 12 elements (Figure 1).

**Figure 2:**
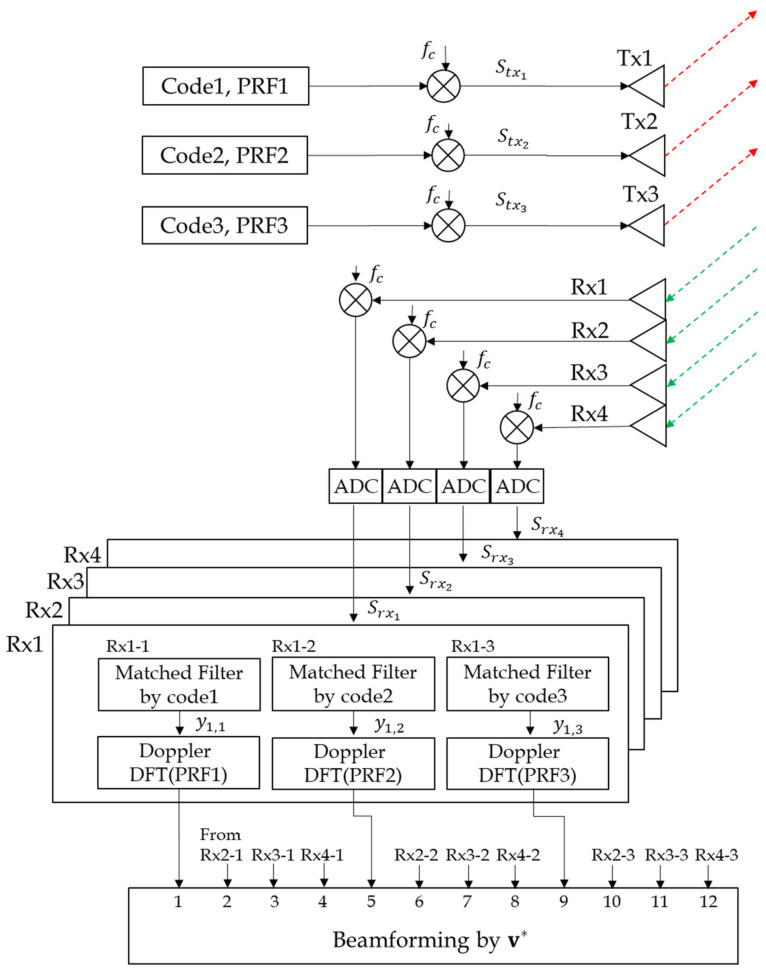
Block Diagram of MIMO Signal processing.

Figure 2 shows the entire block diagram of the proposed MIMO processing. First, each signal from receivers (R × N) is converted to digital data, passed through three matched filters, and then doppler processed. The matched filter is for extracting the matching code and performing pulse compression. MIMO Beamforming is performed by the resulting RD map in the final stage.

Three arrays transmit different waveforms which are represented by

(4) $$\begin{array}{c} S_{tx_{m}}\left(t\right)=\overset{N_{m-1}}{\underset{n=0}{\sum }}C_{m}\left(t-nT_{m,pri}\right)rect\left(\frac{t-nT_{m,pri}}{T}\right)e^{j2\pi f_{c}t}=G_{m}\left(t\right)e^{j2\pi f_{c}t},\;\;\;\;\;m=1,2,3 \end{array}$$

(5) $$\begin{array}{c} rect\left(x\right)=\left\{\begin{array}{c} 1\;\;\;\;\;\;\;\;\left|x\right|\leq \frac{1}{2}\\ 0\;\;\;\;\;\;\;\;\left|x\right|>\frac{1}{2} \end{array}\right. \end{array}$$

where *T* is the pulse width, $C_{m}\;\left(t\right)$ is the modulation code, $T_{m,pri}$ is the pulse repetition interval, $N_{m}$ is the number of pulses of $m^{th}\;$transmitter, and $f_{c}\;$is a carrier frequency. Then the baseband signal $S_{rx_{l}}\left(t\right)$ received by $l^{th}$ array is sum of the transmit signals with the different delay $\tau_{m,l}$ between $m^{th}$ transmitter and $l^{th}\;$receiver.

(6) $$\begin{array}{c} S_{rx_{l}}\left(t\right)=\bar{A}\;\;\overset{3}{\underset{m=1}{\sum }}S_{tx_{m}}\left(t-\tau_{m,l}\right)e^{-j2\pi f_{c}t}+n\left(t\right) \end{array}$$

(7) $$\begin{array}{c} \tau_{m,l}=\frac{2\left(R_{0}-vt\right)-4d\left(m-1\right)\sin\theta -d\left(l-1\right)\sin\theta }{c} \end{array}$$

where $\bar{A}$ is a complex reflective coefficient, $R_{0}$ is an initial distance, v is a target velocity, θ is a target angle, c is the speed of light, and $n\left(t\right)$ is the noise generated through the antenna and transmitter/receiver and assumed to be spatially white and complex Gaussian. By substituting $\tau_{m,l}$ in Equation (7) into Equation (6), the following is obtained.

(8) $$\begin{array}{c} S_{rx_{l}}\left(t\right)=Ae^{jkd\left(l-1\right)\sin\theta }\overset{3}{\underset{m=1}{\sum }}e^{j4kd\left(m-1\right)\sin\theta \;}\cdot G_{m}\left(t-\tau_{m,l}\right)e^{j2\pi f_{d}t}+n_{B}\left(t\right) \end{array}$$

where A is a modified coefficient as $\bar{A}e^{j2\pi f_{c}\left(-2R_{0}/c\right)}$.

The signal processing on each receiver starts with the matched filter to extract each transmit signal. The output of $i^{th}$ matched filter consists of the auto correlation $R_{ii}\left(t\right)$, cross-correlation $R_{im}\left(t\right)$, and the noise $N\left(t\right)$ as follows.

(9) $$\begin{array}{cc} y_{l,i}\left(t\right)=Ae^{jkd\left(l-1\right)\sin\theta }e^{j4kd\left(i-1\right)\sin\theta \;}R_{ii}\;\left(t\right)e^{j2\pi f_{d}t} & \\ & +\overset{3}{\underset{\begin{array}{c} m=1\\ m\neq i \end{array}}{\sum }}e^{4kd\left(m-1\right)\sin\theta \;}\cdot R_{im}\left(t\right)\;e^{j2\pi f_{d}t}+N\left(t\right) \end{array}$$

$$R_{im}\left(t\right)=\int_{t-T/2}^{t+T/2}G_{i}^{*}\left(\tau -t\right)G_{m}\left(\tau -\tau_{m,l}\right)d\tau \;\;$$

The desired output is the first term of the auto correlation and the remaining cross-correlation and noise terms are all interference.

**Figure 3:**
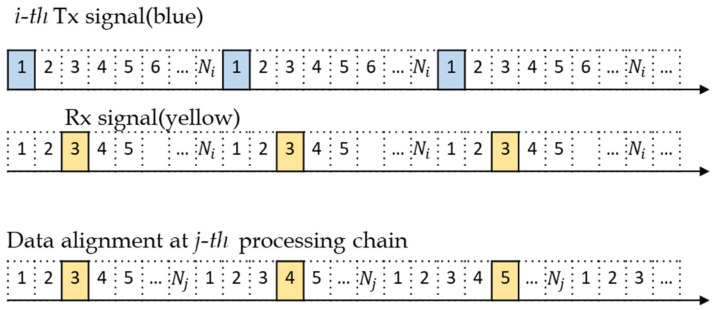
Reflected pulse of the *i*-th transmit signal and data alignment at the *j*-th processing chain when $\Delta_{N}=1\;$and $R_{1}=3$.

Next step is the doppler processing of coherent pulse integration, which is performed for the range bins of the same location among pulse trains. Because transmit waveforms are typically designed to use the same PRF, all reflected signals are in the same range bin. And both the matched code and the mismatched code obtain the same coherent integration gain; thus, no further improvement of the ratio between matched code and mismatched code is made at this stage. In this paper, we suggest a novel method of using different PRFs to suppress the level of cross-correlations further. Using different PRFs is to change the reflection range bins from other transmitters and prevent them from obtaining the coherent integration gain. In other words, the signals from other transmitters cannot be aligned in the same range bin among pulse trains if the PRF is different. Figure 3 shows that the reflected signal from the *i*-th transmitter is not correctly aligned in the *j*-th processing chain supporting another PRI.

### 2.2. Changing PRFs and Constraints

To perform MIMO beamforming in Equation (3), it is important to ensure that the phase information is not changed by different PRFs. Therefore, we first synchronize the start time to align the initial phase and use the same Coherent Integration Time(CPI) to achieve the same doppler resolution. If $N_{i}$ is the number of samples in one pulse repetition interval (PRI) and $M_{i}$ is the number of pulses consisting of one CPI of the $i^{th}$ transmit waveform, then the doppler resolution is determined by

(10) $$T_{B}=N_{i}\times \left(M_{i}-1\right)\;\left(i=1,2,\ldots ,M_{t}\right).$$

This should be constant for all the transmit signals. The maximum value of $N_{i}$ is typically determined by the maximum velocity of the target.

If $N_{i}>N_{j},\;$the reflected pulses of the $i^{th}\;$transmit signal at the $j^{th}\;$processing chain (Rx x-j in Figure 2) are shifted to the right by $\Delta_{N}\times k\;$as the pulse number *k* increases, where $\Delta_{N}=N_{i}-N_{j}$ and $j=1,2,\;M_{t}$. The range bin of the first reflected pulse is the same for all the transmit waveforms because the start time is synchronized. The following pulses are located at

(11) $$R_{k}=R_{1}+\left(k-1\right)N_{i}=R_{1}+\left(k-1\right)N_{j}+\Delta_{N}\left(k-1\right)$$

where $R_{1}$ is the range bin of the first reflected pulse, and $k=1,2,\ldots M_{i}$. If $k=M_{i}$, then the amount of shift is

(12) $$\Delta_{N}\left(M_{i}-1\right)=N_{j}\left(M_{j}-M_{i}\right)=N_{j}\Delta_{M}.\;\;\;$$

This means that the maximum amount of shift is determined by ($M_{j}-M_{i})\;$multiplied by $N_{j}$—the pulse repetition interval. If $\Delta_{M}=M_{j}-M_{i}=1$, then it is less than $N_{j}\;$and

(13) $$\Delta_{N}=N_{i}-N_{j}=\frac{N_{j}}{M_{i}-1}.\;\;\;$$

Therefore, the reflective powers are spread evenly around the entire range bin. If only two transmit signals are used $\Delta_{M}=1$ would be ideal. If $\Delta_{M}>1\;$and $M_{i}-1=n\Delta_{M}$, where *n* is an integer, then

(14) $$\Delta_{N}=\frac{N_{j}}{n}\;\;\left(n<M_{i}-1\right),\;\;\;$$

which means that some of the reflective pulses are concentrated in the same range bin. In addition, when $\Delta_{M}=1$, if $\Delta_{N}$ is greater than the pulse width, the reflective pulses do not overlap in the range bins.

Second, the measurement scale should be the same for all the processing chains. Because the number of pulses is $M_{i}$, an $M_{i}$ point DFT is normally performed, and the measurement scale is $PRF/M_{i}\left(=1/\left[N_{i}\times M_{i}\right]\right)$ if there is no zero padding. However, according to Equation (10), this scale for each waveform is not the same. Therefore, we performed a $k\times \left(M_{i}-1\right)$ point DFT with zero padding, so that the measurement scale is $1/\left[N_{i}\times k(M_{i}-1\right)]=1/kT_{B}$ that is the same for all waveforms, where *k* is an integer greater than 1.

## 3. Simulation Results and Discussion

**Figure 4:**
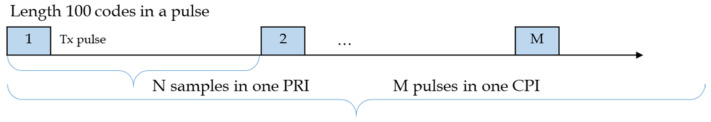
Description of parameters in Table 1.

In this section, we present the simulation results obtained by applying the proposed method. The used waveform of the pulse train is described in Figure 4, and the related parameters satisfying Equation (1) are listed in Table 1.

**Figure 5:**
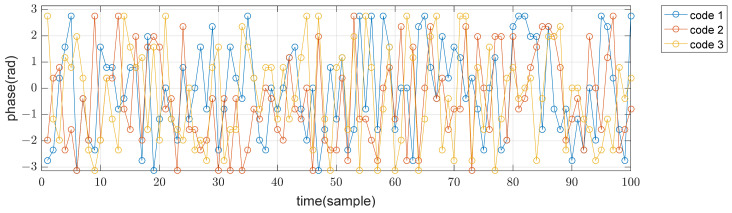
Phase of codes in time domain.

**Figure 6:**
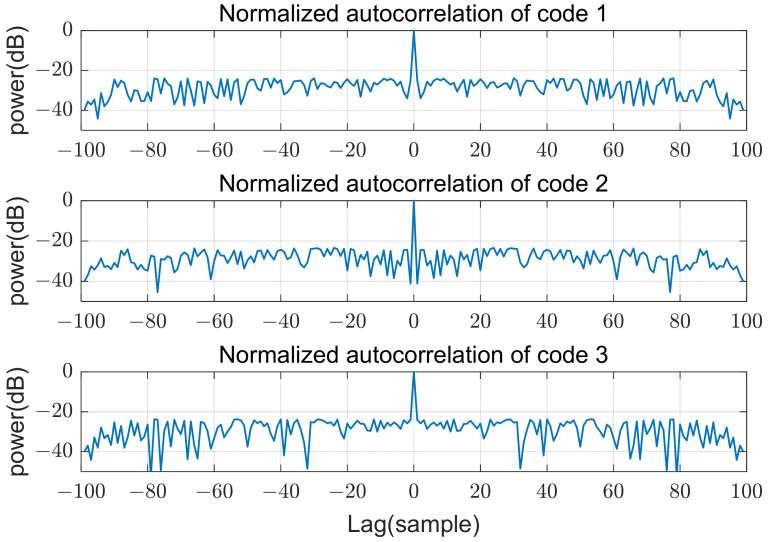
Normalized autocorrelation of codes.

**Figure 7:**
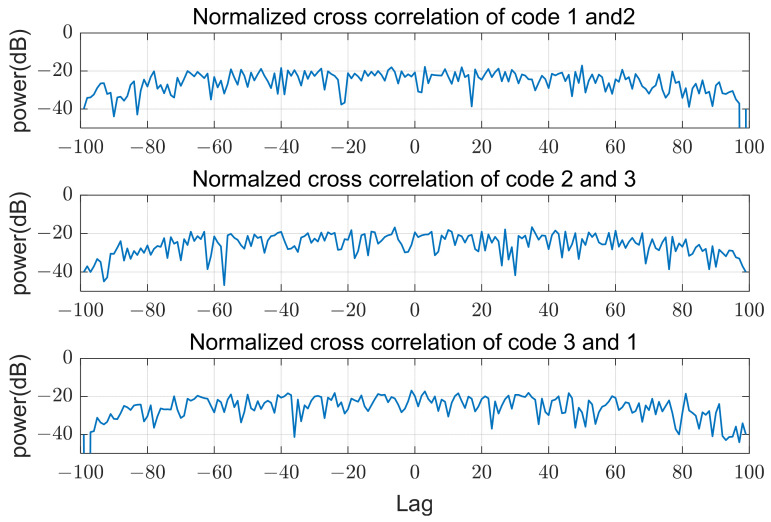
Normalized cross-correlation of codes.

Three transmit arrays and four receive arrays are used for MIMO beamforming, as shown in Figure 1, and the transmit codes in a pulse are poly phase waveforms of length 100 and 16 phase values, which were designed using a simulated annealing algorithm [13]. The cost function of this optimization algorithm is the sum of the peak sidelobe level of autocorrelations and the peak level of cross correlations. Phases of the used codes in time domain are shown in Figure 5. The sidelobe level of autocorrelations and the level of cross-correlations are below −20 dB from the peak, as shown in Figure 6 and Figure 7. In the following sections, $SNR_{i}$ refers to the signal-to-noise ratio at each array input.

### 3.1. Effect of Different PRFs

**Figure 8:**
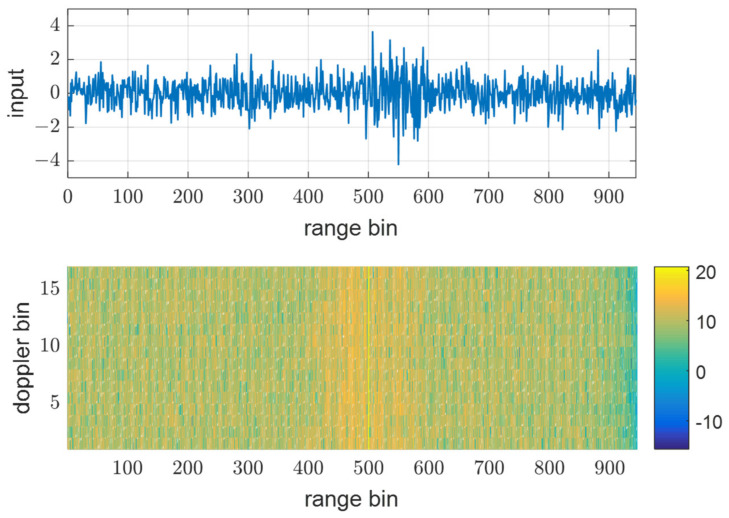
Input data of 10th pulse and matched filter out, $y_{1,3}$ using the same PRF at $SNR_{i}$ = 0 dB.

**Figure 9:**
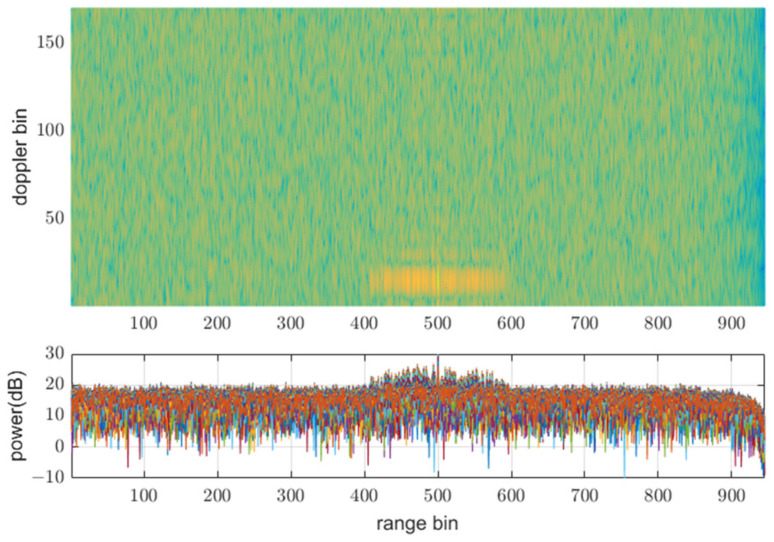
Doppler processing out in Rx1-3 using the same PRF at $SNR_{i}\;$= 0 dB.

**Figure 10:**
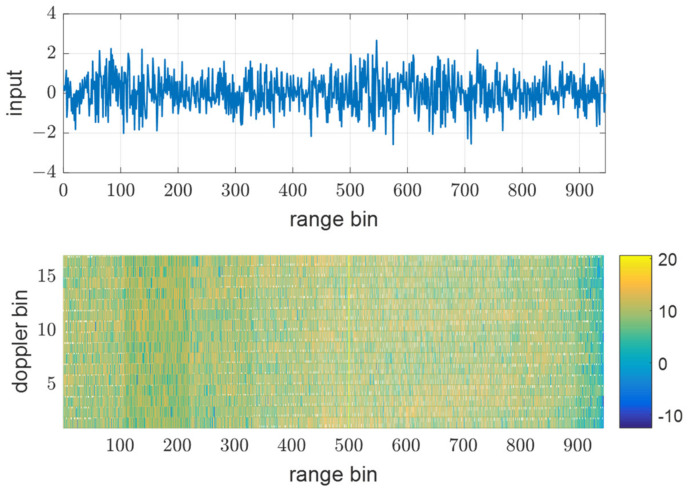
Input data of 10th pulse and matched filter out, $y_{1,3}\;$using the different PRFs at $SNR_{i}$ = 0 dB.

**Figure 11:**
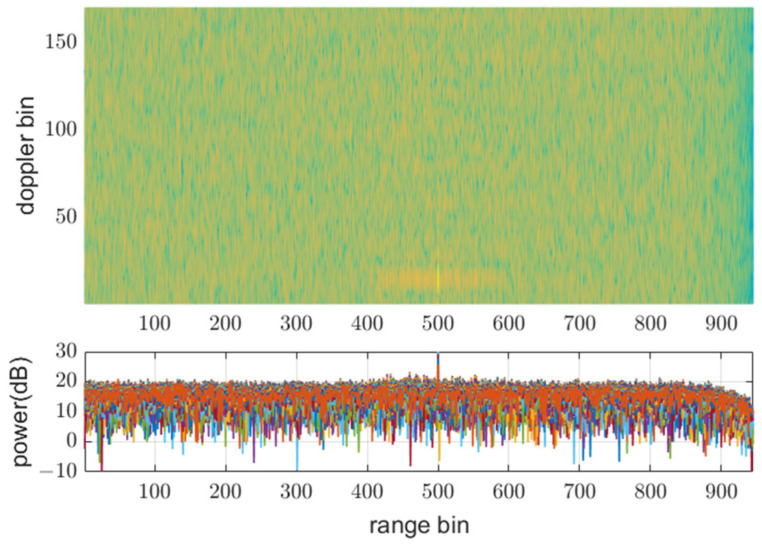
Doppler processing out in Rx1-3 using different PRFs at $SNR_{i}$ = 0 dB.

Figure 8, Figure 9 and Figure 10 show input data and the power of each filter output when using the same PRF(N) of 945 for all transmit waveforms and using different PRFs listed in Table 1, respectively. Figure 8 shows that all the reflective signals are in the same range bins, and the power of mismatched codes is represented by near sidelobe levels. In this case, all the matched and mismatched codes are coherently integrated by the doppler processing, so the relative sidelobe level does not change. Figure 9 shows the RD map (range doppler map) and power over ranges after doppler processing. The doppler processing was performed ten times $\left(M_{i}-1\right)$ point DFT, that is 170 in this case. On the other hand, Figure 10 shows that owing to different PRFs, the power of mismatched codes is distributed across different range bins. Thus, they cannot be integrated coherently by doppler processing, and the sidelobe level around the target decreases, as shown in Figure 11.

**Figure 12:**
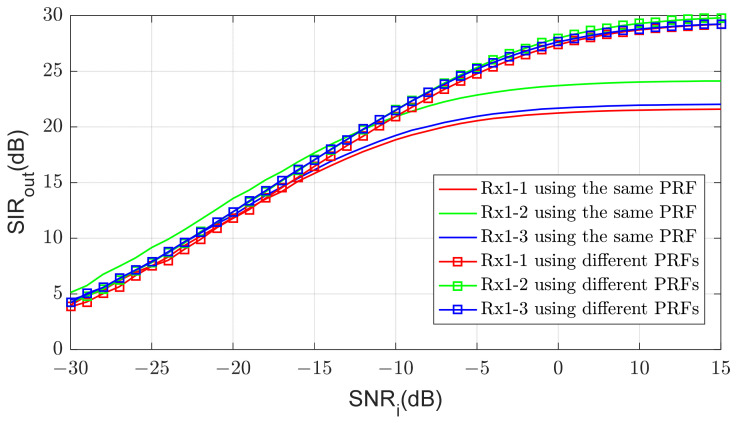
Output ${\mathrm{SIR}}_{\mathrm{out}}$ vs. input ${\mathrm{SNR}}_{i}$.

Figure 12 shows SIR after the doppler processing for varying input SNR. ${\mathrm{SIR}}_{\mathrm{out}}$ is calculated as the ratio of the power of the target signal to the average power of the surrounding 64 bins. When the SNR is small, the noise constitutes most of the interference power, and ${\mathrm{SIR}}_{\mathrm{out}}\;$is almost equal to the SNR improved by processing gain, which is about 34dB in this case. However, as the SNR increases, cross-correlations become dominant in interference power and ${\mathrm{SIR}}_{\mathrm{out}}$ is no longer improved. The limit of ${\mathrm{SIR}}_{\mathrm{out}}$ depends on the cross-correlations and is different at each processing chain.

### 3.2. MIMO Beamforming and Angle Estimation

**Figure 13:**
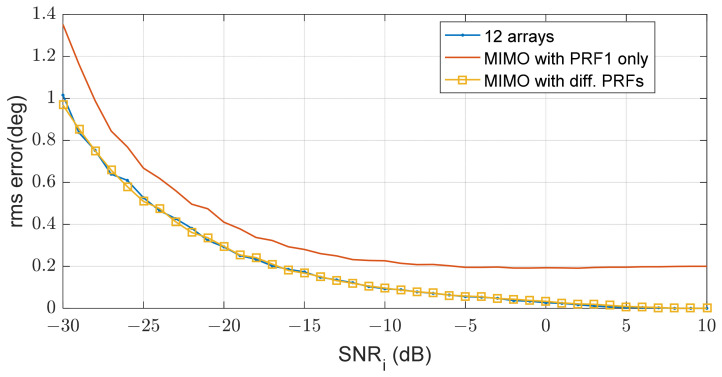
RMS errors according to ${\mathrm{SNR}}_{i}$ for a target at 15 deg.

The remaining interference reduces SIR and eventually affects the angular accuracy of MIMO beamforming. Figure 13 shows the angular estimation error of the target at 15°. The angle estimation is performed by MIMO beamforming according to Equation (3). When all PRFs of the transmit waveforms are the same, there is a limit to the interference level and the angular estimation error is also bounded. However, if the interference level is reduced using different PRFs, the estimation performance is improved. This shows that the RMS error is significantly reduced when using different PRFs compared with using the same PRF and is almost equal to the error of a single 12-array antenna because three transmit signals and four receive arrays synthesizes 12 virtual arrays.

## 4. Conclusions

We propose a method to use different PRFs in a MIMO beamforming system and demonstrate its performance through simulations. To perform MIMO virtual array beamforming, the transmit signals should be separated in each receiver and must be designed as orthogonal waveforms. In radar systems that cannot synchronize the reflected signal, it is very difficult to eliminate the undesirable mismatched codes for all delay times. Moreover, these signals increase with the signal power and limit the performance when the SNR is high. The proposed method spreads the interference signal to other range bins and achieves additional suppression through doppler processing.

Because this method basically spreads the interference signal over the entire range bins, applications in an environment where there are many targets or a lot of clutter will be difficult. However, it is very effective for improving the angular accuracy of the MIMO radar for a small number of aerial targets.

## Figures and Tables

**Table 1:** Waveform parameters.

Number of Tx.	N (PRI)	M (Pulses)	Code Length
1	1080	15	100
2	1008	16	100
3	945	17	100

## Data Availability

Not applicable.
